# Tumor markers in clinical practice: General principles and guidelines

**DOI:** 10.4103/0971-5851.56328

**Published:** 2009

**Authors:** S. Sharma

**Affiliations:** *Department of Surgical Oncology, Amrita Institute of Medical Sciences & Research Centre, Ernakulam - 682026, Kerala, India*

**Keywords:** *Tumor markers*, *serum tumor markers*, *early diagnosis*, *malignancy*

## Abstract

Tumor markers are assuming a growing role in all aspects of cancer care, starting from screening to follow-up after treatment, and their judicious application in clinical practice needs a thorough understanding of the basics of pathophysiology, techniques of identification or testing, reasons for out-of-range levels of tumor markers, as well as the knowledge of evidence of their role in any given malignancy. These are, at the most, just an adjunct to diagnosis, and establishing a diagnosis on the basis of tumor markers alone (especially a single result) is fraught with associated pitfalls because of the problem of nonspecificity. In reality an ideal tumor marker does not exist. Detection can be done either in tissue or in body fluids like ascitic or pleural fluid or serum. Clinical uses can be broadly classified into 4 groups: screening and early detection, diagnostic confirmation, prognosis and prediction of therapeutic response and monitoring disease and recurrence. In addition to variable sensitivity and specificity, the prevalence of a particular malignancy may be a major determinant in the application of a particular test as a screening tool. Serum levels, in certain situations, can be used in staging, prognostication or prediction of response to therapy. Monitoring disease is, perhaps, the most common clinical use of serum tumor markers. Rising trend in serum levels may detect recurrence of disease well before any clinical or radiological evidence of disease is apparent ("biochemical recurrence"). Sampling should ideally be repeated after 5-6 half-lives of the marker in question (or the marker with the longest half-life if multiple markers are being considered); but if found elevated, the next sampling after 2-4 weeks, for additional evidence, may be justified.

## INTRODUCTION

Current clinical practice in oncology has a growing impetus on early diagnosis, proper prognostication and (of late) screening for malignancy in asymptomatic groups. Tumor markers are assuming a growing role in all aspects of cancer care, starting from screening to follow-up after treatment. Important clinical decisions are increasingly likely to be made on the basis of these results, whether for diagnosis, screening, prediction or treatment monitoring.[1]

The first known attempt to find markers for malignancy was made 2000 years ago and is described in an Egyptian papyrus, where breast cancer was distinguished from mastitis.[2] Incidentally the first tumor marker in modern medicine was identified by Bence-Jones, who in 1846 detected a heat precipitate in samples of acidified urine from patients suffering from "Mollities osseum".[2] In 1965, Gold *et al.,* isolated a glycoprotein molecule from specimens of human colonic cancer and thus discovered the first "tumor antigen," later identified as carcino-embryonic antigen (CEA).[3]

Today there are literally hundreds of tumor markers, although their clinical utility or application is, of course, a different issue!

Tumor markers include a variety of substances like cell surface antigens, cytoplasmic proteins, enzymes, hormones, oncofetal antigens, receptors, oncogenes and their products.[4] There have been numerous attempts to broaden the definition to accommodate the rapidly expanding set of identified tumor markers and include the following:

1. Substances present in, or produced by, a tumor itself or produced by host in response to a tumor that can be used to differentiate a tumor from normal tissue or to determine the presence of a tumor based on measurements in blood or secretions.[4,5]
2. A molecule, a process or a substance that is altered quantitatively or qualitatively in precancerous or cancerous conditions, the alteration being detectable by an assay.[6]
3. Biochemical indicators of the presence of a tumor.[7] However, in common clinical practice, the term usually refers to a molecule that can be detected in plasma or other body fluids.

## IDEAL TUMOR MARKER

Only a few tumor markers have stood the test of time and entered in the diagnostic or management algorithms for clinicians.[1,2,5]

The three most important characteristics of an ideal tumor marker are (a) it should be highly specific to a given tumor type, (b) it should provide a lead-time over clinical diagnosis and (c) it should be highly sensitive to avoid false positive results. Additionally, the levels of the marker should correlate reliably with the tumor burden, accurately reflecting any tumor progression or regression, along with a short half-life allowing frequent serial measurements. The test used for detection should be cheap for screening application at mass level and should be of such nature as to be acceptable to the target population [Table 1]. In reality an ideal tumor marker does not exist.

**Table 1:** Characteristics of an ideal tumor marker [5]

Characteristics	Remarks
Highly specific	Detectable only in one tumor type
Highly sensitive	Non-detectable in physiological or benign disease states
Long lead-time	Sufficient time for alteration of natural course of disease
Levels correlate with tumor	Prognostic and predictive utility of
burden	the tumor marker
Short half-life	Frequent serial monitoring of the marker levels after 5-6 half lives
Simple and cheap test	Applicability as screening test
Easily obtainable specimens	Acceptability by target population

## MOLECULAR BASIS OF TUMOR MARKERS

Genetic alteration in a tumor cell affects directly or indirectly the gene expression pattern of the tumor cell or the surrounding tissue.[7] These genetic alterations can be reflected at various levels [Table 2], from viral genomic incorporation to genetic defects, forming the molecular basis of tumor markers.[8]

**Table 2:** Molecular basis of tumor markers [8]

Levels of classification	Examples
DNA	
Epigenetic	Promoter Hyper-methylation, e.g., GSP1, DAP in lung cancer; p15, p16 in liver cancer
Endogenous	Mutations, e.g., NADH dehydrogenase 4
Mitochondrial genetic	(ND4) in urine in bladder cancer
Oncogene	Mutation, e.g., *K-ras* in pancreatic cancer; micro-satellite alterations in head and neck cancers
Exogenous viral	EBV in NPC, Burkitt′s lymphoma; HPV in cervical cancer
RNA	
Cell based	Tissue-specific markers, e.g., PSA mRNA
endogenous	in prostate cancer, cytokeratin 20 mRNA in breast cancer
Cell free	Circulating mRNA, e.g., Tyrosinase mRNA in melanoma
Exogenous viral	Viral RNA, e.g., EBV-coded RNA in NPC
Translational protein	
Native protein	PSA in prostate cancer, CEA in colonic
(Conventional markers)	cancer
Glycan	Aberrant glycosylation, e.g.,monosialytactec AFP in HCC

AFP: Alfa fetoprotein; CEA: Carcinoembryonic antigen; EBV: Epstein-Barr virus; HCC: Hepatocellular carcinoma; HPV: Human papilloma virus; mRNA: Messenger RNA; NPC: Nasopharyngeal carcinoma; PSA: Prostate-specific antigen

## METHODS OF DETECTION

The methods of detection can be classified into 6 major groups [Table 3].[2,5,6,9,10] The most common method in use today is serological enzyme assays.

**Table 3:** Methods of detection of tumor markers[2,5,6,9,10]

Serology	Enzyme assays
Immunological	Immuno histo chemistry
	Radio immuno assay
	Enzyme-linked immuno sorbent assay
Flow cytometry	
Cytogenetic analysis	Fluorescent *in-situ* hybridization
	Spectral karyotyping
	Comparative genomic hybridization
Genetic analysis	Sequencing (automated)
	Reverse transcription
	Gel electrophoresis
	DNA micro-array analysis
Proteomics	Surface-enhanced laser desorption/Ionization

Immunological detection usually relies on monoclonal antibodies that specifically bind to epitopes on tumor markers and are in turn tagged for identification with dyes in immunohistochemistry *(IHC)*, radioactive tags in radio-immuno assay *(RIA)*, or enzymes in enzyme-linked immunosorbent assay *(ELISA)*.[2,5,6,10,11] Alternatively, in a suspension, flow cytometry can analyze the presence and percentage of antibody-tagged cells.[9–11] These methods are highly sensitive and can detect quantities in the nanogram to picogram range (10^–6^to 10^−9^g). Of these, the most commonly used technique today is IHC. Uses of IHC in oncology include categorization of undifferentiated malignant tumors, categorization of leukemias and lymphomas, determination of site of origin of metastatic tumors and detection of molecules of prognostic or therapeutic significance (e.g., Estrogen/progesterone receptors (ER/PR) in breast cancer).[2,5–7,10]

## CLASSIFICATION AND USES

Tumor markers can be detected either in tissue (tissue tumor markers; for example, in solid tumors, lymph nodes, bone marrow or circulating tumor cells in the blood) or in body fluids like ascitic or pleural fluid or serum (serological tumor markers).[9,10] Tissue tumor markers are of prime importance to a diagnostic pathologist, while the serological tumor markers are more often used by a clinician and will be discussed in more detail in this review.

Many classification schemes exist based on differences in origin, structure, biological function or their relationship to the event in tumor growth or formation [Table 4].[1,11]

**Table 4:** Classification scheme for tumor markers [2]

Category	Subcategory	Examples
Oncofetal antigens		AFP, CEA
Hormones		Catecholamines
		Calcitonin, β-hCG
Glycoproteins		CA 125, CA 15-3, CA
		19-9, CA 72-4, PSA
Metabolites		VMA, HIAA
Tumor-associated	Viral antigens	Polyoma, SV 40
antigens		
	MHC-related	H-2 k antigen
	antigens	
	Enzymes	PAP, NSE, PLAP
	Oncogene products	c-myc, c-erbB2
	Cytogenetic	Philadelphia
	products	chromosome
Tumor-associated	Proteins	Immunoglobulins,
markers		β-2M
	Enzymes	Lactate
		dehydrogenase,
		alkaline phosphatase,
		pteridines, pterines
	Acute-phase	C-reactive protein,
	proteins	ferritin
	Inflammatory	ESR, viscosity
	makers	
Ultrastructural	Intermediate	Desmin, vimentin
components	filament components

AFP: Alfa fetoprotein; CEA: Carcinoembryonic antigen; ESR: Erythrocyte sedimentation rate; HIAA: Hydroxy indole acetic acid; NSE: Neuron-specific enolase; PAP: Prostatic acid phosphatase; PLAP: Placental alkaline phosphatase; PSA: Prostate-specific antigen; SV: Simian virus; VMA: Vanillmandelic acid

Clinical application of tumor markers can be broadly classified into 4 groups: Screening and early detection, diagnostic confirmation, prognosis and prediction of therapeutic response and monitoring disease and recurrence.[5,7,9,10] Some of the recommended uses of tumor markers in routine clinical practice are summarized in Table 5.

**Table 5:** Common clinical uses of some tumor markers[2 5 9]

Malignancy	Tumor marker (s)	Tumor marker detection method	Suggested roles
Adrenal carcinoma	Steroids, Catecholamines	Serology	D
Breast	CA 15-3, CA 27.29	Serology / Tissue IHC	M, R
	ER / PR / Her-2neu	Tissue IHC	RT
Carcinoid	5-HIAA	Serology / Urine	D
Colorectal, stomach, pancreas	CEA, CA 19-9	Serology / Tissue IHC	P, M
Choriocarcinoma	β-hCG	Serology / Tissue IHC	D, P, M
Germ cell tumors	AFP,β-hCG	Serology / Tissue IHC	D, P, M
	LDH, PLAP (Seminoma)	Serology	P, M
Hepatoma	AFP	Serology / Tissue IHC	S, D, P, M
Lymphomas	LDH	Serology	D, P
	Cytogenetic alterations	Genetic analysis	D
Melanoma	Tyrosinase	Serology	D
Myeloma	Immunoglobulins	Serology	D, P
Ovarian	CA 125	Serology / Tissue IHC	M, D, R
Prostate	PSA	Serology / Tissue IHC	S, M, D, P
Sarcomas	Cytogenetic alterations	Genetic analysis	D
Thyroid	Thyroglobulin	Serology / Tissue IHC	S, M
	Calcitonin (medullary carcinoma)	Serology	S, M, P

M = Monitoring; R = Recurrence; S = Screening; P = Prognosis; D = Diagnosis; RT = Response to therapy; AFP: Alfa fetoprotein; β-hCG: Beta human chorionic;gonadotropin; CA: Carbohydrate antigen; CEA: Carcinoembryonic antigen; ER: Estrogen receptor; HIAA: Hydroxy indole acetic acid; LDH: Lactate dehydrogenase; PLAP: Placental alkaline phosphatase; PR: Progesterone receptor; PSA: Prostate-specific antigen

Although extremely appealing, the concept of screening a large apparently healthy population for occult tumors using a tumor marker and thereby enabling early therapeutic intervention is not currently possible because no test yet devised is 100 percent specific and many tumor markers may be elevated in benign conditions [Table 6].[2,7,10,11]

**Table 6:** Some benign conditions associated with rise in tumor markers[2]

Marker	Associated nonmalignant conditions
AFP	Viral hepatitis, liver injury, IBD, pregnancy
β-hCG	Testicular failure, marijuana smokers, pregnancy
CEA	Smokers, IBD, hepatitis, cirrhosis, pancreatitis,gastritis
CA 125	Peritoneal irritation, endometriosis, pelvic inflammatory disease, hepatitis, pregnancy
PAP / PSA	Prostatitis, benign prostatic hyperplasia

AFP: Alfa fetoprotein; β-hCG: Beta human chorionic gonadotropin; CA: Carbohydrate antigen; CEA: Carcinoembryonic antigen; IBD: Inflammatory bowel disease; PAP: Prostatic acid phosphatase; PSA: Prostate-specific antigen

Importance and use of a particular tumor marker may change depending upon the clinical scenario, ranging from initial presentation to differential diagnosis to recurrence.[2] Thus, for example, while tissue tumor markers like Cytokeratin, smooth muscle antigen (SMA), etc., may be extremely useful in categorization of malignancy, they are of no use in prognosis or monitoring; on the other hand, markers like *Ki-67 *(proliferation index) may help in prognostication or choice of therapy but have no role in the diagnostic arena.

A large body of literatures exists on guidelines suggested for clinical application in various malignancies by professional bodies like American Society of Clinical Oncology,[12,13] Canadian Task Force,[29] American Association for Clinical Chemistry,[1] etc., but the only tumor marker that finds a place in any screening algorithm is prostate-specific antigen (PSA).

Attempts to improve the sensitivity and/ or specificity of tumor markers have led to combination of tumor markers with other procedures (e.g., combination of Carbohydrate antigen (CA) 125 with ultrasonography for early detection of ovarian malignancy) or to refining the evaluation criteria for tumor markers (e.g., PSA density or PSA velocity or age-specific PSA cut off ranges for early detection of prostate cancer). However, these have either not stood the rigorous evaluation of randomized trials or have still not received widespread approval of professional clinical organizations.[1,12–14]

In addition to variable sensitivity and specificity of tumor markers, the prevalence of a particular malignancy may be a major determinant in the application of a tumor marker as a screening tool. A stark example of this effect is seen in China, where serum alfa fetoprotein (AFP) has been successfully used as a screening tool for primary hepatoma in endemic regions in contrast to its failure in rest of the world.[15]

By and large, tumor markers cannot be construed as primary modalities for the diagnosis of cancer, mainly because of the lack of sufficiently high specificity and sensitivity. Their main utility in clinical medicine is as a laboratory test to support the diagnosis or in follow-up of patients being treated for malignancy.[10] However, since the prevalence of disease is likely to be higher in diagnostic situations, tumor markers, in conjunction with other diagnostic modalities, are helpful in differentiating between benign and malignant diseases. A good example can be CA 125, which at levels more than 95 IU/mL, especially in postmenopausal women with adnexal mass on radiological imaging, is virtually confirmatory of ovarian malignancy. This may not be true in premenopausal ladies, wherein conditions like pelvic inflammatory disease, endometriosis, etc., represent examples of elevation of CA 125 levels in benign conditions.[16] Another example is the role of AFP in classification of germ cell tumors. Seminomatous germ cell tumors are typically associated with increase in Beta human chorionic gonadotropin (β-hCG) in 10% to 30% of cases, but an increased AFP level is never seen. A case of germ cell tumor with an elevated AFP level is treated as a case of non-seminomatous variant irrespective of histopathological classification.[17] It is worth pointing out here that the use of tumor markers in differential diagnosis is gaining more and more acceptance for histopathological classification using tissue tumor markers.

Tumor marker levels, in certain situations, reflect tumor burden in the body and hence can be used in staging, prognostication or prediction of response to therapy.[5] Malignancies where serum tumor markers are included in the staging protocols include testicular germ cell tumors (LDH, AFP, β-hCG)[18] and lymphoma (LDH). [19] Tumor marker levels can also be used to evaluate the response to therapy, although there may be an initial delay before the tumor marker levels register a decline following treatment.[5,20,21]

Monitoring disease, perhaps, constitutes the most common clinical use of serum tumor markers.[5] Markers usually increase with progressive disease, decrease with remission and do not change significantly with stable disease. Tumor marker kinetics is generally more important than individual values.[20,21] Rising tumor marker levels may detect recurrence of disease well before any clinical or radiological evidence of disease is apparent ("biochemical recurrence").

Determination of risk usually involves genetic probes or tools to evaluate any specific genetic abnormality or mutation noted to confer an increased risk of a particular malignancy. Examples of such abnormalities would include carriers of Philadelphia chromosome for hematological malignancies; and BRCA 1 or 2 genes, which confer a higher risk of breast or ovarian malignancies.[22]

## RECOMMENDATIONS FOR ORDERING TUMOR MARKER TESTS

It is imperative to remember that though an aggressive investigative approach may be warranted on the basis of raised tumor marker values, treatment cannot be initiated without undisputable documentation (often histological) of the disease.[23] There may be instances such as initiation of therapy on the basis of β-hCG levels in cases of choriocarcinoma, but these are few, exceptional and clearly defined.

A single value or test is unreliable in itself.[6] It is noteworthy that in most situations, elevations of markers in nonmalignant diseases are often transient, whereas elevations associated with cancer either remain constant or continuously rise. Ordering serial testing can help detect falsely elevated levels due to transient elevation. Establishing a diagnosis on the basis of tumor markers (especially a single result) is fraught with associated pitfalls because of the problem of nonspecificity. [24-31]

Knowledge of the assay method is important in interpretation of either an abnormal value or a serial change in tumor marker values.[20,29,30] Various methods of detection have their own specific cut off values and sensitivities.[20,21] Thus, for any set of serial values to be meaningful, they have to come from the same assay methods and preferably from the same laboratory. In certain situations of so-called biochemical recurrences, it is always useful to go back to the laboratory to confirm this before beginning (at times) a frustrating search for the elusive recurrence.

It is imperative to be certain that the marker in question was, in fact, elevated before relying on it for monitoring disease activity, the reason being that none of the tumor markers are 100% sensitive (may not be elevated in some cases).[2,5,7,10] In tumors with multiple raised markers measured prior to definitive therapy, the marker showing highest elevation should be used for follow-up.[7,10] If in a given case, tumor markers were not evaluated in the pretreatment setting, it is advisable to use multiple markers for monitoring in the post-therapy setup.[5,7,10]

Tumor marker kinetics should always be factored before repeating the tests. Too frequent estimation of the tumor marker may misrepresent the course of the disease due to distribution and elimination kinetics. As a general guideline, the time interval between serial determinations should be 3 months; but in case of an abnormal value, a repeat estimate can be ordered within 2 to 4 weeks irrespective of the initial reading. The success of surgical removal of a tumor as determined by tumor marker concentrations is ideally ascertained after a period not less than 5-6 half-lives, to allow tumor marker levels to make a plateau or fall to normal. This period may be even longer in case of treatment with chemotherapy or radiotherapy, wherein the therapeutic effects themselves are manifested after a lag period.[20,21]

Patient characteristics affect the tumor marker values to a significant degree. The anticipated fall in levels may not be evident in situations where the metabolism or excretion of the tumor marker is altered, like in patients with renal or liver disease, depending on whether the tumor marker is removed through glomerular filtration or metabolized by the liver. For example, serum CEA is often elevated in patients with liver diseases because the metabolism of CEA by the diseased liver is subnormal. False tumor marker elevation is also known to occur in other confounding situations like smoking, ethanol consumption, COPD, etc., especially if there has been a recent change in habits.[5]

Usually multiple tumor markers are associated with individual malignancy [Table 7]; vice versa, individual tumor markers may be associated with various malignancies [Table 8]. [28] Thus the use of multiple markers based on the combination pattern for the selected malignancy will improve sensitivity and specificity of the detection.[32,33] However, tumor markers that run parallel to each other, when correlated with tumor behavior, should not be selected for this purpose.[32–34]

**Table 7:** Selected examples of malignant diseases with associated tumor markers[27]

Malignant disease	Major marker	Other markers
Bone cancer	Alkaline phosphatase	Bence Jones protein, serum calcium
Breast cancer	CA 15-3	CEA, calcitonin, β-hCG, LASA-P, Prolactin
Carcinoid tumors	Chromogranin A	Histamine, ADH, Bradykinin
Cervical cancer	SCC-A	AG-4 antibodies, CA 125, CEA, TPA
Colorectal cancer	CEA	CA 19-5, CA 19-9, CA 72-4, CK-BB, NSE
Gastric carcinoma	CA 72-4	CA 19-9, CA 50, CEA, ferritin, CK-BB, β-hCG, LASA-P, pepsinogen II, prothrombin
HCC	AFP	CEA, ferritin, ALP, γ-glutamyl transpeptidase
Insulinoma	Insulin	C-peptide, IGF-1–binding protein
Leukemia	TdT	ALP, β2M, ferritin, LDH, myelin basic protein, adenosine deaminase, PNP
Lung cancer	NSE	ACTH, CK-BB, calcitonin, CA 72-4, CEA, AFP, ferritin, LASA-P, TPA
Lymphoma	β2M	TdT, Ki-67, LASA-P
Medullary thyroid cancer	Calcitonin	NSE
Multiple myeloma	Immunoglobulin heavy and light chain	Bence Jones protein, β2M, IgA
Non-seminomatous	AFP	β-hCG, LDH
testicular tumor		
Ovarian carcinoma	CA 125	Inhibin, AFP, CEA, CK-BB, b-hCG, galactosyl transferases, LDH, TPA
Pancreatic carcinoma	CA 19-9	CA 19-5, CA 50, CA 72-4, CEA, CK-BB, ADH, ALP, γ-glutamyl transpeptidase, PAP
Pheochromocytoma	Metanephrine	Chromogranin A, plasma catecholamines
Prostate carcinoma	PSA	PAP, ALP, CEA, CK-BB, TPA
RCC		Rennin, erythropoietin, IL-_4_, prostaglandin A, VA 15-3, PTH, NSE, prolactin

ACTH: Adrenocorticotropic hormone; ADH: Antidiuretic hormone; AFP: Alfa fetoprotein; ALP: Alkaline phosphatase; b2M: Beta 2 microglobulin; CA: Carbohydrate antigen; CEA: Carcinoembryonic antigen; CK-BB: Creatine kinase BB isoenzyme; HCC: Hepatocellular carcinoma; IGF-1: Insulin-like growth factor 1; IL: Interleukin; LASA-P: Lipid- associated sialic acid P; LDH: Lactate dehydrogenase; NSE: Neuron-specific enolase; PAP: Prostatic acid phosphatase; PNP: Purine nucleoside phosphorylase; PSA: Prostate- specific antigen; PTH: Parathyroid hormone; RCC: Renal cell carcinoma; SCC-A: Squamous cell carcinoma antigen; TdT: Terminal deoxynucleotidyl transferase; TPA: Tissue polypeptide antigen

**Table 8:** Selected examples of serologic tumor markers and malignant diseases associated with each [27]

Tumor marker	Associated malignancy	
	Primary	Other malignancies
Oncofetal antigens		
AFP	Primary HCC	Teratoblastomas of the ovary and testes
CEA	Colorectal carcinoma	Various carcinomas
Hormones		
β-hCG	Choriocarcinoma	Testicular cancers (non-seminomatous), trophoblastic tumors
Calcitonin	Medullary carcinoma	Cancer of the thyroid, liver cancer, renal cancer
Metanephrines	Pheochromocytoma	Neuroblastoma, ganglioneuromas
Chromogranin A	Pheochromocytoma, neuroblastoma	MEN, small-cell lung cancer, carcinoid tumors
IGF- 1	Pituitary cancer	Insulinoma
Glycoproteins		
CA 15-3	Breast cancer	Various carcinomas
CA 19-9	Pancreatic and gastric carcinomas	Various carcinomas
CA 72-4	Gastric carcinoma	Various carcinomas
CA 125	Ovarian carcinoma	Various carcinomas
Isoenzymes		
PSA	Prostate cancer	
NSE	Small-cell lung carcinoma	Neuroblastoma, kidney tumors
Cellular components/products		
LASA-P		Various carcinomas, leukemia, lymphoma, Hodgkin's disease
SCC-A		Squamous cell carcinoma of the uterus, cervix, lung, and head and neck
TAG 72	Gastric carcinoma	Colorectal, lung, pancreatic and ovarian cancers
Immunoglobulins	Multiple myeloma	Gammopathies

AFP: Alfa fetoprotein; β-hCG: Beta human chorionic gonadotropin; CA: Carbohydrate antigen; CEA: Carcinoembryonic antigen; HCC: Hepatocellular carcinoma; LASA-P: Lipid- associated sialic acid P; MEN: Multiple endocrine neoplasia; NSE: Neuron-specific enolase; PSA: Prostate-specific antigen; SCC-A: Squamous cell carcinoma antigen

Nonspecific tumor markers are a good choice for monitoring disease activity. Although nonspecific tumor markers, by definition, have a poor sensitivity, nevertheless their concentrations are sensitive to any alterations in tumor volume. They are usually inexpensive and simple to measure. For example, lipid-associated sialic acid P (LASA-P) can be quantified with a simple, rapid and inexpensive calorimetric procedure, and its serum concentration is closely parallel to the serum concentrations of many tumor markers of higher specificity in various malignancies.[34]

An important interfering factor to be considered before any interpretation is presence of a hook effect.[30] This is especially true if the value of a tumor marker does not correlate to the clinical situation. Hook effect is an inherent flaw of certain methods of detection (specifically immunoassay) due to which the serum tumor marker levels may be reported to be falsely low if the concentration rises above a particular level.[30,35] Testing the same sample at two separate dilutions (no dilution and 1:10 dilution), if the clinical picture warrants, will detect this phenomenon. In the presence of a hook effect, the 10-fold diluted sample will yield a value that is higher than the value from the original specimen extrapolated to the same dilution.

Additionally, ectopic tumor markers can be a source of diagnostic dilemma.[28] Malignant cells, by definition, have lost inherent control of the synthetic and multiplicative machinery, and the cell becomes autonomous. Autonomic expression of various unrelated genes (for the given tumor tissue type) is the reason for the expression of ectopic tumor markers in advanced malignant diseases [Table 9]. Ectopic tumor markers denote dedifferentiation by indicating activation of unrelated genes and, in other words, are associated with poorer prognosis or metastases. For example, elevated levels of AFP may be detected in patients with gastrointestinal tract malignancy with metastases to liver, although the liver function tests may be normal.

**Table 9:** Ectopic tumor markers[27]

Ectopic tumor marker	Primary tumor site
AFP	Gastrointestinal, renal, breast, bladder and ovary carcinoma
Calcitonin	Carcinoma of lung, islet cell, carcinoid, breast and ovary; medullary carcinoma; pheochromocytoma
Chromogranin A	For endocrine tumors (medullary thyroid carcinoma, anterior pituitary adenoma,pancreatic islet-cell carcinoma)
β-hCG	Gastric and pancreatic carcinomas,hepatoma, ovarian adenocarcinoma,germinal-cell tumors of the testis
Thyroglobulin	WD thyroid carcinoma

AFP: Alfa fetoprotein; β2M: Beta 2 microglobulin; WD: Well differentiated

## INTERPRETATION

According to guidelines published by *Working Group on Tumor Marker Criteria,* interpretation should take into account the therapy status of the patient.[36-38]

If the patient is under active treatment or has received treatment in the recent past, changes in marker levels may reflect the clinical progression of the disease. Partial remission is defined as a decrease in marker levels by at least 50%; and progressive disease, as an increase in marker levels by at least 25%, on the basis of the concept that tumor load is related to changes in serum tumor marker levels.

An important caveat put forth by this working group in the use of tumor markers in monitoring response to therapy is that *"a complete remission cannot be determined by tumor marker levels, but if tumor marker levels are elevated, the clinical decision of complete remission based on conventional methods should be considered incorrect unless an explanation for the presence of an elevated level is given"*.[37,38]

However, if no therapy has been given in the recent past, during monitoring for a malignancy, a linear rise in three consecutive samples (i.e., over a two time intervals) on a log scale should be noted before a recurrence can be established.[37,38] Sampling for tumor marker levels should ideally be repeated after 5-6 half-lives of the marker in question (or the marker with the longest half-life if multiple markers are being considered); but if found elevated, the next sampling after 2-4 weeks, for additional evidence, may be justified.[36]

Physiological influences that need to be considered in interpreting the results include effects of aging and menopause; metabolism and route-of-elimination kinetics of the tumor marker; coexisting disease, like renal or liver failure; hormonal imbalances, like hyperthyroidism/ hypothyroidism; etc.[20,21] Life-style influences on tumor marker values include states like smoking (increases levels of CEA, AFP, etc.), alcoholism (altered liver and renal parameters), obesity (hormonal imbalances, altered steroidal metabolism in peripheral fat), etc.

## CONCLUSIONS

The use of tumor markers in clinical oncology has increased tremendously with rapid expansion of techniques of detection and identification of new markers in recent times, a trend that continues to grow as technology progresses and our understanding about our body and the disease processes increases. However, such use is not without its pitfalls; in fact, injudicious application of tumor markers is fraught with risks of mistreatment (under-treatment or over-treatment) and its consequences.

Of the numerous tumor markers identified, described and extensively researched upon, only a handful of them are used in routine clinical practice; and even of these, only a few have established consensus guidelines for use in day- to-day care of patients.

With the explosion in the pool of knowledge, clinical application of tumor markers in the field of oncology represents a classical example of "losing sight of the forest for leaves of a tree." It is indeed unfortunate that the emphasis (in medical education, research and literature) has been on more and more extensive and in depth knowledge of individual tumor markers, their pathophysiology, genetic origin, etc., rather than basic broad understanding and its application in routine clinical practice.

Judicious application of tumor markers to clinical practice needs a thorough understanding of the basics of pathophysiology, the techniques of identification or testing, reasons (in cases of both benign and malignant tumors) for out-of-range levels of tumor markers, as well as the knowledge of evidence of their role in any given malignancy.
